# Caffeine and Selective Adenosine Receptor Antagonists as New Therapeutic Tools for the Motivational Symptoms of Depression

**DOI:** 10.3389/fphar.2018.00526

**Published:** 2018-06-01

**Authors:** Laura López-Cruz, John D. Salamone, Mercè Correa

**Affiliations:** ^1^Àrea de Psicobiologia, Universitat Jaume I, Castellón de la Plana, Spain; ^2^Behavioral Neuroscience Division, University of Connecticut, Storrs, CT, United States

**Keywords:** adenosine receptors, dopamine, caffeine, antidepressants, anergia, fatigue, anxiety

## Abstract

Major depressive disorder is one of the most common and debilitating psychiatric disorders. Some of the motivational symptoms of depression, such anergia (lack of self-reported energy) and fatigue are relatively resistant to traditional treatments such as serotonin uptake inhibitors. Thus, new pharmacological targets are being investigated. Epidemiological data suggest that caffeine consumption can have an impact on aspects of depressive symptomatology. Caffeine is a non-selective adenosine antagonist for A_1_/A_2A_ receptors, and has been demonstrated to modulate behavior in classical animal models of depression. Moreover, selective adenosine receptor antagonists are being assessed for their antidepressant effects in animal studies. This review focuses on how caffeine and selective adenosine antagonists can improve different aspects of depression in humans, as well as in animal models. The effects on motivational symptoms of depression such as anergia, fatigue, and psychomotor slowing receive particular attention. Thus, the ability of adenosine receptor antagonists to reverse the anergia induced by dopamine antagonism or depletion is of special interest. In conclusion, although further studies are needed, it appears that caffeine and selective adenosine receptor antagonists could be therapeutic agents for the treatment of motivational dysfunction in depression.

## Major Depression Disorder: Symptomatology and Current Treatment

Major depression disorder (MDD) is one of the most debilitating disorders in the world, and the most commonly diagnosed according to the World Health Organization. The Diagnostic and Statistical Manual in its last edition (DMS-5) defines this disorder as a set of symptoms including: depressed mood, decreased interest or pleasure in almost all activities nearly every day, appetite changes (changes in body weight), sleep disturbances, feelings of worthlessness or guilt, diminished ability to concentrate or indecisiveness, psychomotor agitation or retardation and fatigue or loss of energy (American Psychiatric Association, 2013).

Although depression is typically defined as an affective disorder, it also appears that some symptoms such as psychomotor retardation, fatigue, and loss of energy are related to deficits in motivation, specifically in activational aspects of motivation. Motivated behavior is directed toward or away from particular stimuli, but it also is characterized by a high degree of activity, effort, vigor, and persistence (Salamone and Correa, 2002, 2012). People with depression commonly show profound activational impairments, such as lassitude, listlessness, fatigue, and anergia (low self-reported energy) that affect their motivation (Tylee et al., 1999; Stahl, 2002). In fact, among depressed people, energy loss and fatigue are the second most commonly reported symptoms, only behind depressed mood itself (Tylee et al., 1999), and depressed patients with anergia are more common than patients with anxiety related symptoms (Tylee et al., 1999; Drysdale et al., 2017). Furthermore, in depressed patients “lack of energy” was the factor that correlated to problems with fatigability, inability to work, and psychomotor retardation, loading most strongly onto a second order general depression factor (Gullion and Rush, 1998). Many people with MDD have fundamental deficits in reward seeking, exertion of effort, and effort-related decision making that do not simply depend upon any problems that they may have with experiencing pleasure (Treadway et al., 2009). Lack of energy is the symptom most highly correlated with a lack of social function in depressed patients, and is correlated with various work-related impairments such as days in bed, days of lost work, and low work productivity (Swindle et al., 2001). In addition, this cluster of symptoms can be highly resistant to treatment (Stahl, 2002); they are the best predictors of lack of remission after antidepressant drug treatment (Stahl, 2002; Gorwood et al., 2014).

## Pharmacological Treatments for the Activational Symptoms in Depression

The severity of effort-related motivational symptoms in depression is related to problems with social function, employment absence, and treatment outcomes (Tylee et al., 1999; Stahl, 2002). Patients with high scores in psychomotor retardation also have longer duration of illness, an earlier age of onset, and more depressive episodes (Calugi et al., 2011; Gorwood et al., 2014). These symptoms are a predictor of delayed response to treatment with either interpersonal psychotherapy or selective serotonin (5-HT) reuptake inhibitor pharmacotherapy (Frank et al., 2011), often remaining as residual symptoms even in patients in remission (Stahl, 2002; Fava et al., 2014; Gorwood et al., 2014).

Most of the present treatment strategies for MDD focus on drugs that block the inactivation (i.e., inhibitors of enzymatic breakdown or uptake) of the monoamine neurotransmitters 5-HT and norepinephrine (NE). The classical antidepressants include monoamine oxidase inhibitors (MAOIs), which affect one of the major catabolic enzymes for monoamines (Quitkin et al., 1979), and drugs that inhibit uptake of one or more monoamines (Feighner, 1999; Yıldız et al., 2002). Although 5-HT and NE reuptake inhibitors have become the most frequently prescribed medications for MDD, they fail to complete symptom remission in 40–60% of all patients (Rush and Trivedi, 1995; Fava et al., 2014), and it is widely accepted that at least 20% of all depressed patients do not respond adequately to most antidepressant drugs (Crown et al., 2002). Many common antidepressants, including 5-HT transport inhibitors such as fluoxetine, are relatively ineffective at treating anergia and fatigue, and in fact, can induce or exacerbate these symptoms (Padala et al., 2012; Stenman and Lilja, 2013; Fava et al., 2014).

Interestingly, some clinical studies suggest that drugs that inhibit dopamine (DA) transport, such as the catecholamine uptake inhibitor bupropion, are relatively more effective than 5-HT uptake inhibitors for treating effort-related motivational symptoms (Rampello et al., 1991; Stahl, 2002; Demyttenaere et al., 2005; Pae et al., 2007). Furthermore, individual differences in behavioral traits can differentiate between depressed patients that are more responsive to bupropion (i.e., motivated, achievement-oriented, active, exercise-oriented people) vs. fluoxetine (people with mood problems, irritability, and rumination) (Bell et al., 2013). Stimulant drugs that are not considered to be antidepressants in the classical sense, such as methylphenidate and modafinil, have been shown to increase energy and motivation in depressed patients (Zisook et al., 2006). Thus, clinical studies, together with preclinical investigations (e.g., Salamone et al., 2006, 2007; Salamone and Correa, 2012; Argyropoulos and Nutt, 2013; Heath et al., 2015), have led to the suggestion that DA systems and related circuits are particularly involved in effort-related motivational symptoms.

## Adenosine Receptors Co-Localization with DA Receptors

In addition, another possible therapeutic target for the anergia component of depression is adenosine receptors. Adenosine is a neuromodulator in the central nervous system (CNS) that plays an important role in the regulation of synaptic transmission and neuronal excitability (Cunha, 2001; Sebastião and Ribeiro, 2009). Several subtypes of adenosine receptors are expressed in the brain, with A_1_ and A_2A_ G-protein-coupled receptors being the most abundant (Jacobson and Gao, 2006; Fredholm et al., 2011). A_2A_ receptors are expressed at high levels in the striatum and olfactory bulbs and tubercle (Fredholm et al., 2011), but also in areas such as amygdala, hippocampus or prefrontal cortex (Cunha et al., 1994; Pandolfo et al., 2013; Simões et al., 2016). Adenosine A_1_ receptors have a higher widespread distribution in the brain, with a somewhat higher concentration in hippocampus (Schwarzschild et al., 2006). All these regions are involved in the regulation of complex processes such as cognition, motivation, and emotion (Hauber and Sommer, 2009; Salamone and Correa, 2012) that seem to be altered in MDD.

**FIGURE 1:**
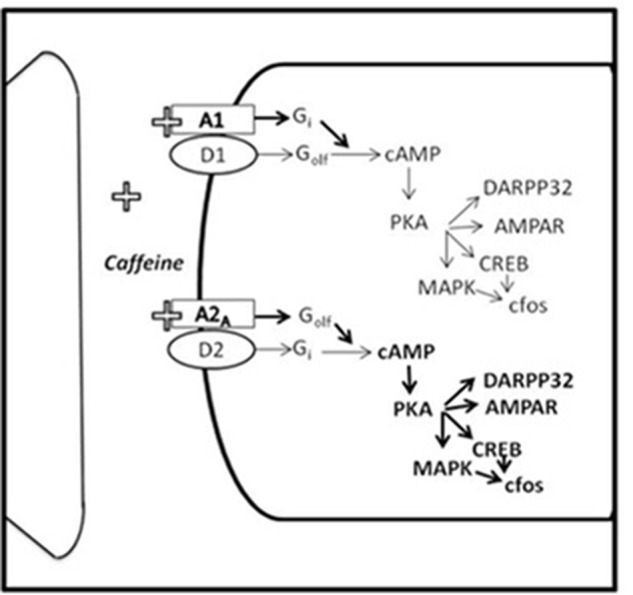
Impact of caffeine on the functional interaction between adenosine and DA receptors. A_1_R and A_2A_R, adenosine A_1_ and A_2A_ receptors; D_1_, DA type 1 receptor; D_2_, DA type 2 receptor (adapted from Ferré, 2008).

The spatial distribution of adenosine receptors within the brain (Fredholm et al., 2011) allows a wide range of effects, including modulation of other neurotransmitter systems (Cunha, 2001). Thus, adenosine A_2A_ receptors are highly expressed postsynaptically in DA rich areas such as neostriatum and accumbens (Acb) (Johansson and Fredholm, 1995; Johansson et al., 1997; DeMet and Chicz-DeMet, 2002; Rebola et al., 2005). In fact, it has been demonstrated that in these areas, there is a functional interaction between DA D_2_ and adenosine A_2A_ receptors (see **Figure 1**), which are co-localized on enkephalin-containing medium spiny neurons and converge onto the same signal transduction pathways in an antagonistic way (Ferré et al., 1997, 2008; Fuxe et al., 2003; Ferré, 2008; Beggiato et al., 2014). Similarly, A_1_ and D_1_ receptors antagonistically interact on substance P-containing medium spiny neurons (Ferré et al., 1997, 2008).

The behavioral significance of this interaction has frequently been studied in the context of neostriatal motor functions and pathologies (Ferré et al., 1997; Correa et al., 2004; Collins et al., 2010). Thus, selective A_2A_ receptor antagonists are being tested in clinical trials for pathologies involving DAergic dysfunctions such as Parkinson disease, and positive results indicate that they can be used as adjuvant therapies (Hung and Schwarzschild, 2014). Caffeine actions on A_1_ and A_2A_ adenosine receptors (Ferré, 2008), has promoted its study as an alternative preventive or therapeutic tool for parkinsonian symptoms (Prediger, 2010). Moreover, within the last years, the motivational significance of DA-adenosine receptor interactions has become apparent with regard to processes such as behavioral activation, and effort-related decision-making impaired in depression or other pathologies (Salamone et al., 2006; Salamone and Correa, 2009).

In the present review, we focus on studies that assessed the effect of adenosine antagonists on different aspects of depression in humans, as well as in animal models. Special emphasis will be placed on motivational/psychomotor symptoms induced by DA depletions and studies related to DA-adenosine interactions in pathological symptoms related to effort-related decision-making.

## Caffeine Consumption and Depression

Caffeine is a naturally occurring methylxanthine that acts mainly as a non-selective A_1_ and A_2A_ adenosine receptor antagonist (Fredholm et al., 1999). This methylxanthine is found in common beverages including coffee, tea, soft drinks, and products containing cocoa, as well as a variety of medications and dietary sources (Barone and Roberts, 1996; Wikoff et al., 2017), ranking as one of the most commonly consumed dietary ingredients throughout the world (Heckman et al., 2010). Daily intake of caffeine among consumers in United States is about 280 mg, and higher intakes are estimated in some European countries (Barone and Roberts, 1996). Caffeine is typically consumed in order to increase alertness, arousal and energy (Malinauskas et al., 2007). Its consumption has been related to changes in cognitive performance and mood in normal population (Smith, 2013; Pasman et al., 2017). However, it enhances performance more in fatigued than well-rested subjects (Lorist et al., 1994; Childs and de Wit, 2008).

There are very few studies on the relation between caffeine consumption and depression-related symptoms, and in many cases, its use is related to self-medication patterns. Some of these studies focus on the role of caffeine as a drug that prevents depression, while others discuss caffeine as a possible treatment for existing depression. Thus, secondary analyses of large epidemiological databases with similar number of men and women indicate that in non-clinical samples that do not work, consumption of caffeine (around 150 mg/day as average) was associated with a reduced risk of depression (Smith, 2009). Also, in a longitudinal study in women free from depressive symptoms at baseline, high levels of caffeine consumption (>550 mg/day) was negatively correlated with the appearance of depressive symptoms (Lucas et al., 2011). In fact, the relative risk for depression was highest for those women with lower caffeine consumption (<100 mg/day) (Lucas et al., 2011). However, in women with multiple sclerosis high doses of caffeine (>400 mg/day) increased the prevalence of MDD (Patten et al., 2000). Moreover, in non-clinical samples, although caffeine consumption at moderate doses was related with decreases in suicide risk (Kawachi et al., 1996; Tanskanen et al., 2000; Lucas et al., 2014), excessive consumption (750 mg/day) was correlated with a higher risk of suicide (Kawachi et al., 1996; Tanskanen, 1997; Lucas et al., 2014). Thus, from the present studies, it seems that intermediate levels of caffeine consumption (300–550 mg/day) produce beneficial effects in non-clinical populations, but not in people with some neurological pathologies. Higher doses will have negative effects, even in non-clinical populations.

Multiple reports have lent support to the idea that depressed people could use caffeine as self-medication. It has been reported that psychiatric patients show a relatively high degree of caffeine consumption compared to the normal population (Greden et al., 1978; Leibenluft et al., 1993; Rihs et al., 1996). This appears to be particularly true in patients that have experienced depressive symptoms (Leibenluft et al., 1993). Different profiles of patients (i.e., with alcohol dependence, seasonal affective disorder, and people with MDD) have been shown to have higher levels of caffeine consumption after experiencing depressive symptoms (as shown by the Hamilton Rating Scale for depression) (Hamilton, 1967; Leibenluft et al., 1993). Specially, among youth with depression, there generally is higher caffeine consumption that in the general population (Whalen et al., 2008). Moreover, the degree of caffeine consumption seems to be a predictor of improvement of somatic symptoms (fatigue among them), and hostility in depressed patients medicated with fluoxetine (Worthington et al., 1996), suggesting that caffeine could be an effective co-treatment for some of the symptoms of depression. However, it is important to note that, at high doses or in people with susceptibility, caffeine is also known to increase anxiety and insomnia (for a review Temple et al., 2017), two side effects that can contribute to worsen MDD. At high doses, however, it has been demonstrated that caffeine may not act as an adenosine receptor antagonist, and other underlying mechanisms seem responsible of its negative effects (for a recent review Fredholm et al., 2017).

## Impact of Caffeine on Energy/Fatigability and Behavioral Activation in Humans

A wide range of studies demonstrate that caffeine can increase alertness and subjective energy, and also reduce fatigue (Johnson et al., 1990b, 1991; Yu et al., 1991; Smith et al., 1992, 1997; Lieberman, 2001), thus acting as an ergogenic substance. Caffeine has been demonstrated to increase feelings of efficiency, self-confidence, motivation to work (Fredholm et al., 1999), and to improve psychomotor performance (Rees et al., 1999). The behavioral effects of caffeine can be influenced by the baseline arousal levels and also by the nature of the task requirements. It has been argued that the most evident effects of caffeine on fatigue would be expected in situations of low arousal or high fatigue, or in tasks placing high demands on controlled processing (Bachrach, 1966; Lieberman et al., 1987). In fact, beneficial effects of caffeine have been observed in people in low states of alertness, such as after benzodiazepines administration (Johnson et al., 1990b), sleep loss (Childs and de Wit, 2008; Paech et al., 2016), when the person has a cold (Smith et al., 1997), or when the experiment is done in the early morning (Smith et al., 1992). In addition, a broad range of studies have reported effects of caffeine withdrawal on different markers of motivation using descriptors such as fatigue, decreased energy or vigor, lethargy, amotivation for work, etc. (for a review see Juliano and Griffiths, 2004). For example, in controlled studies, after 10 days of high levels of caffeine consumption (1,250 mg/day), withdrawal results in increased subjective ratings of headache, sleepiness, laziness, and fatigue, as well as decreased alertness, activation and vigor (Juliano et al., 2012). Abstinence from intermediate doses in daily coffee and cola consumers (579 mg/day), increased ratings of drowsy/sleepy, fatigue/tired, lazy/sluggy/slow-moving, decreased ratings of active/energetic/excited and motivation to work, and impaired performance on psychomotor tasks (Liguori and Hughes, 1997). Even at low quantities (100 mg/day, in a controlled study), caffeine withdrawal increased ratings of lethargy, fatigue, tiredness, and sluggishness, and decreased ratings of energy, motivation and urge to work (Griffiths et al., 1990).

## Effect of Caffeine and Adenosine Antagonists on Classic Animal Models of Depression

Preclinical studies have been trying to elucidate the effect of caffeine and selective adenosine antagonists on classical animal models of depression (El Yacoubi et al., 2001). Two of the classic tests for the assessment of antidepressant properties of different substances in rodents are the forced swim test (FST) and the tail suspension test (TST). In the FST animals develop an immobile posture in an inescapable cylinder filled with water (Porsolt et al., 1977; Petit-Demouliere et al., 2005). The TST is based on the observation that a mouse suspended by the tail shows alternating periods of agitation and immobility (Steru et al., 1985). Classical antidepressants reduce immobility time in these paradigms, which have become the gold standard to evaluate antidepressant effects of multiple drugs or to show depressive symptoms induced by behavioral manipulations (Armario and Nadal, 2013). In this regard, learned helplessness has been considered as one of the causes for developing depression in vulnerable individuals that suffer stressful life events. This phenomenon is reproducible in animal models in which the depressive-like state is induced either by chronic uncontrollable and unpredictable stressors (CUS), typically electrical foot-shock (Overmier and Seligman, 1967), but also by chronic mild stress (CMS) induced by irregular exposure to a combination of different types of stressors over a period of weeks (Willner, 2005). In addition, animals that develop learned helplessness show a disruption in escape performance as well as decreases in weight gain, increased immobility in the FST or TST, and reduced locomotion, all symptoms associated to some degree with depression (Seligman, 1972). After the administration of substances with antidepressant properties, animals exposed to CUS or CMS display escape-directed behaviors, reducing time of immobility (Porsolt et al., 1977; Steru et al., 1985).

All these tests and manipulations have been used to study the therapeutic properties of caffeine and selective adenosine antagonists or genetic deletion of adenosine receptors in rodents. In one of the seminal papers, Porsolt et al. (1977) demonstrated that an acute dose of caffeine reduced immobility time in the FST in Sprague-Dawley rats. In later studies, this effect has been confirmed using other strains of rats and mice, after acute or repeated administration of a broad range of doses (3.0–30.0 mg/kg) and using diverse animal tests (FST, TST) (Kulkarni and Mehta, 1985; Kaster et al., 2004, 2015; Robles-Molina et al., 2012; Kale and Addepalli, 2014; Minor and Hanff, 2015; Szopa et al., 2016). In accordance with the effects of caffeine, adenosine A_2A_ receptor antagonists have also been effective in these tests. Thus, SCH58261 and istradefylline (KW6002) reduced total immobility time in both the TST and the FST in mice (El Yacoubi et al., 2001). SCH58261 also reduced immobility time in a selectively bred ‘helpless’ mice strain in the TST (El Yacoubi et al., 2001). Moreover, A_2A_ receptor knockout (A_2A_KO) mice showed reductions in immobility time compared to wild type (WT) animals in both tests (El Yacoubi et al., 2001).

Using the learned helplessness model for inducing depressive symptoms, it has been demonstrated that acute doses as well as chronic administration of caffeine can reduce the impact of CUS (Woodson et al., 1998; Hunter et al., 2003; Minor et al., 2008; Pechlivanova et al., 2012; Kaster et al., 2015). Thus, pharmacological or genetic blockade of A_2A_ receptors not only prevented but also reversed CUS-induced behavioral and physiological signs of depression such as decreased weight gain, increased corticosterone levels, escape behavior impairments in a shuttle box, increased immobility time in the FST and TST, increased anxiety, and decreased locomotion and spatial reference memory (Kaster et al., 2015). However, caffeine only reverted the deficits of reference memory but did not reverse mood-related alterations (Machado et al., 2017) in mice genetically selected to display ‘depressive’-like symptoms (El Yacoubi et al., 2003). Consistent with these findings, mice that received the selective A_2A_ receptor antagonist istradefylline, as well as the constitutive A_2A_KO mice, were protected from the CUS-induced behavioral impairments in the FST, TST, and memory tests (Kaster et al., 2004), suggesting a key role for A_2A_ receptors in acute and chronic stress-induced depressive effects.

**FIGURE 2:**
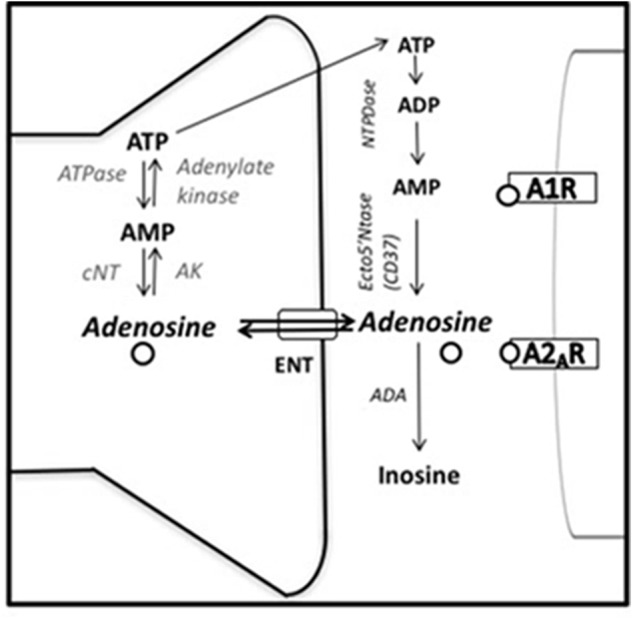
Adenosine synthesis and metabolism. ADA, adenosine deaminase; AK, adenosine kinase; A_1_R and A_2A_R, adenosine A_1_ and A_2A_ receptors; cNT, cytosolic endo-nucleotidase; ENT, equilibrative nucleoside transporter; Ecto5′Ntase (CD73), 5′-ectonucleotidase; NTPDase, nucleoside triphosphate dephosphorylase (adapted from Ruby et al., 2011).

Based on these results some researchers have focused on adenosine receptor antagonists, including caffeine, as tools to reverse behavioral impairments induced by pharmacological manipulations of the adenosine system (Kulkarni and Mehta, 1985; Minor et al., 1994a, 2008; Woodson et al., 1998; Hunter et al., 2003; Pechlivanova et al., 2012). Thus, high doses of acutely administered adenosine (50.0–100.0 mg/kg, intraperitoneally IP) (Kulkarni and Mehta, 1985), or its analoge 1-chloroadenosine (2.0 mg/kg, IP) induce immobility in the FST in mice, and caffeine as well as theophylline (8.0 mg/kg, IP), reversed this effect (Kulkarni and Mehta, 1985). Theophylline, is a psychoactive methylxanthine found in tea and other substances, and is also a metabolite of caffeine that acts as a non-selective adenosine antagonist for A_1_/A_2A_ receptors as well (Gu et al., 1992). Increases of adenosine in the central nervous system have been also associated with escape deficits in the inescapable shock paradigm (Kulkarni and Mehta, 1985; Minor et al., 1994b; Woodson et al., 1998; Minor and Hanff, 2015). Thus, it has been demonstrated that intraventricular (ICV) administration of NBTI [S-(4-nitrobenzyl)-6-theoinosine], an equilibrative nucleoside transporter (ENT) blocker that increases extracellular adenosine levels blocking its reuptake (see **Figure 2**), impaired escape latency in rats (Jacobson et al., 1992; Noji et al., 2004) at the same level that rats preexposed to 100 inescapable tail shocks, and potentiates escape impairments produced by 50 inescapable tail shocks (Minor et al., 2008). Moreover, ICV administration of erytrho-9(2-hydroxy/3/nonyl adenine (ENHA), a selective adenosine deaminase (ADA) inhibitor which blocks adenosine metabolism, mimicked the effect of inescapable shock (Woodson et al., 1998). This manipulation increases the concentration of extracellular adenosine by blocking the major degradation pathway. Low doses of caffeine reversed escape deficits induced by EHNA (Woodson et al., 1998). The reversal effects of caffeine appear to be specific to actions on adenosine receptors, and not as a general stimulant psychomotor effect, since amphetamine exacerbated the behavioral impairments induced by inescapable shocks (Minor et al., 1994a). In addition, caffeine reversed the escape deficit produced by a bilateral injection of glutamate into the prefrontal cortex of rats (Hunter et al., 2003). This escape deficit induced by glutamate in the prefrontal cortex has been also associated with enhanced adenosine (Petty et al., 1985), since increases in glutamate are counterbalanced by an increase in adenosine production and release (Deckert and Gleiter, 1994; Kerkhofs et al., 2018).

Caffeine has also been used to enhance the effect of monoaminergic antidepressants (especially 5-HT/NE uptake inhibitors) that are being used in clinical practice, and have been demonstrated to reduce immobility in classical animal tests of depression. Thus, caffeine at low doses that do not have an effect on their own can potentiate the effects of desipramine, imipramine, duloxetine, fluoxetine and paroxetine, in animals tested on the FST (Robles-Molina et al., 2012; Kale and Addepalli, 2014; Szopa et al., 2016). In addition, a low dose of caffeine can also improve the effect of bupropion (a DA/NE uptake inhibitor), potentiating extracellular levels of DA and NE (Kale and Addepalli, 2014).

## Impact of Adenosine Antagonists on Behavioral Activation: Preclinical Studies

Tasks measuring behavioral activation and effort-based functions have been suggested as potential animal models for the motivational symptoms of depression (Salamone, 2007; Markou et al., 2013; Salamone et al., 2016). Thus in the animal literature, as in the human data, there are studies showing how caffeine and selective adenosine antagonists affect the willingness to work depending on the demands of the task. In operant tasks with different work demands, caffeine and theophylline produced rate-dependent effects on lever pressing to obtain palatable food in rats (Randall et al., 2011). Moderate doses of caffeine and theophylline (5.0–20.0 mg/kg) increased responding on the low task with low response demands; a fixed interval 240 s (FI-240 s) schedule. However, higher doses (10–40 mg/kg) decreased responding on a fixed ratio 20 (FR20), schedule that typically generates high rates of responding (Randall et al., 2011). A_2A_ receptor antagonists increased lever pressing in the low effort-demanding task (FI-240 s) but did not suppress the high effort task (FR20) in the dose range tested. In fact, there was a tendency for istradefylline to increase FR20 responding at a moderate dose. A_1_ antagonists failed to increase lever-pressing rate, but decreased FR20 responding at higher doses. These results suggest that the work potentiating effects of methylxanthines are mediated by their actions on adenosine A_2A_ receptors, while their A_1_ receptor antagonistic action could be mediating the suppressant effects.

Progressive ratio (PR) schedules, which require gradually increasing work output, have been also employed to explore the effect of caffeine on motivation to work for sucrose or food in rats and monkeys (Buffalo et al., 1993; Brianna Sheppard et al., 2012; Retzbach et al., 2014). Acutely and chronically moderate doses of caffeine (5–25 mg/kg) elevated PR lever pressing for sucrose (Brianna Sheppard et al., 2012; Retzbach et al., 2014). Caffeine had no effect on inactive lever presses suggesting that this increase was not due to an increase in general motor activity (Retzbach et al., 2014). Recently, our laboratory has demonstrated that caffeine has differential effects on PR performance depending on baseline individual differences (SanMiguel et al., 2018). Caffeine (5.0–10.0 mg/kg) increased responding for a solution containing sucrose in low baseline responders, but decreased lever pressing (10.0–20.0 mg/kg) in high responders (SanMiguel et al., 2018). However, in rhesus monkeys intravenous (IV) caffeine (10.0 mg/kg) decreased percent of task completed, and breakpoint in a PR for palatable food (Buffalo et al., 1993), possibly because this dose directly administered in the blood stream resulted in higher levels in the brain.

Thus, from studies in rats and monkeys it seems that high doses of caffeine have an impairing effect on performance in tasks that evaluate willingness to work for a reinforcer if performance is already high. Methylxanthines can help to increase work output when the requirement of the task is low. However, selective A_2A_ receptor antagonists seem to be beneficial independently of the baseline performance, as demonstrated also in goals directed tasks (Li et al., 2016).

## Effort Based Decision-Making Deficits Induced by Interference with DA Function: Potential Therapeutic Role of Adenosine Antagonists

Activational aspects of motivation (i.e., vigor, persistence, work output) are highly adaptative because they enable organisms to overcome obstacles or work-related response costs that separate them from significant stimuli (Salamone and Correa, 2002, 2012; van den Bos et al., 2006). An important feature of adaptive behavior, in the face of work-related challenges, is effort-related decision making. Regularly, organisms must make cost/benefit analyses in which they weigh the value of a stimulus relative to the cost of obtaining it (Salamone et al., 2007, 2016). People with MDD show impairments in estimation, anticipation, and recall of reinforcing stimuli (Pizzagalli, 2014), and also show a reduced likelihood of selecting high effort activities in human tasks of effort-related decision making (Treadway et al., 2012; Yang et al., 2014).

Extensive animal data have demonstrated that Acb DA is a key mediator of effort-based decision-making processes (for a review see Salamone et al., 2016). Interference with DA transmission biases behavior toward less valued rewards that involve less effort and less activity. In these preclinical studies addressing the effort-related decision-making process, animals are given a choice between a more valued reinforcer that can only be obtained by engaging in a more demanding (higher effort) activity vs. a low effort/low value option. One such procedure is a T-maze task that provides an effort-related challenge by having a vertical barrier in the arm with the higher reward density (HD) vs. an arm that contains a lower density of reward (LD) and has no barrier (Salamone et al., 1994; Cousins and Salamone, 1996; Cousins et al., 1996; Mott et al., 2009; Pardo et al., 2012). In this procedure, rodents choose to climb the barrier to get more reward in 90% of the trials, once they have been trained (Cousins and Salamone, 1996; Pardo et al., 2012). In operant tasks animals are given a choice between lever pressing for the more preferred reward (in FR5 or PR schedules) vs. approaching and consuming a less preferred reinforcer that is concurrently freely available in the chamber (Salamone et al., 1991; Randall et al., 2012; Pardo et al., 2015). When tested on the concurrent FR5/free reward choice task, rats typically spend most time pressing the lever for the preferred reward and less consuming freely available food or fluids (Salamone et al., 1991; Pardo et al., 2015). In contrast, rats tested on the PR/chow choice task show more individual variability, and tend to disengage more readily from the PR lever pressing component because of the increasing work requirement (Randall et al., 2012, 2014). Research with these concurrent choice tasks has shown that interference with DA transmission via DA depletions or DA receptor antagonism typically biases rodents toward the low effort-low reward option (Salamone et al., 1991; Salamone and Correa, 2009; Worden et al., 2009; Pardo et al., 2012; Randall et al., 2012, 2014; Yohn et al., 2015).

Using these effort related choice procedures, it has been demonstrated that the catecholamine depleting agent and vesicular transport inhibitor (VMAT-2) tetrabenazine (TBZ), reduces selection of high effort alternatives, but animals compensate by increasing the amount of free less preferred reinforcer consumed (Nunes et al., 2013; Randall et al., 2014; Pardo et al., 2015; Yohn et al., 2015, 2017). TBZ depletes monoamines, with its greatest impact being upon striatal DA (Pettibone et al., 1984; Tanra et al., 1995; Nunes et al., 2013). TBZ is used as a therapeutic drug to treat Huntington’s disease patients, and it induces symptoms of depression in humans, including fatigue (Frank, 2010; Guay, 2010; Rodrigues et al., 2017). TBZ has also been used in the FST and TST rodent models of depression (Kent et al., 1986; Wang et al., 2010). Although the effort-related effects of TBZ are attenuated by the DA uptake blocker bupropion (Nunes et al., 2013; Randall et al., 2014; Yohn et al., 2015) which is been used as an antidepressant, other classical drugs for the treatment of depression such as the 5-HT uptake inhibitors fluoxetine and citalopram, and the NE uptake inhibitor desipramine, failed to reverse the effects of TBZ, and higher doses even led to further behavioral impairments (Yohn et al., 2015, 2016b,c).

In addition to DA, adenosine also is involved in these effort related decision-making processes (Farrar et al., 2007, 2010; Hauber and Sommer, 2009; Mott et al., 2009; Salamone and Correa, 2009). Microinjections of adenosine A_2A_ receptor agonists into the Acb produced effects on instrumental behavior and effort-related choice that resembled those produced by Acb DA receptor antagonism or depletion (Font et al., 2008; Mingote et al., 2008). In addition, considerable evidence indicates that DA D_2_ and adenosine A_2A_ receptors interact to regulate effort-related functions (Salamone and Correa, 2009, 2012). Thus, adenosine A_2A_ receptor antagonists were able to reverse the shift in choice toward a low effort alternative induced by administration of the D_2_ antagonists haloperidol and eticlopride (Farrar et al., 2007, 2010; Mott et al., 2009; Salamone et al., 2009; Worden et al., 2009; Pardo et al., 2012, 2013). Moreover, A_2A_ KO mice were resistant to the effects of haloperidol on performance of the T-maze barrier task (Pardo et al., 2012). Recently, it has been demonstrated that A_2A_ KO mice are also resistant to the anergia inducing effects of D_2_ antagonism in a paradigm in which animals can choose between exercising on a much preferred running wheel or sedentary consuming sweet food (Correa et al., 2016). In contrast, adenosine A_1_ antagonists were ineffective at reversing the effort-related effects of either the D_1_ receptor antagonist ecopipam or the D_2_ receptor antagonist eticlopride (Salamone and Correa, 2009; Nunes et al., 2010; Pardo et al., 2012).

The therapeutic effect of caffeine and theophylline on effort-related choice behavior after the administration of D_2_ antagonists has also been reported in rats tested on the concurrent FR5/chow feeding choice task. Caffeine partially attenuated the effects of haloperidol, increasing the lever pressing and decreasing the free chow intake in haloperidol-treated rats (Salamone et al., 2009) and the same pattern of results were observed in a more recent study in which caffeine reversed the anergia-like effect induced by TBZ in an adapted version of the T-maze task with RW (Correa et al., 2016) increasing the time running (effortful option) and decreasing the time spent eating free available sweet pellets (sedentary option) (López-Cruz et al., 2018). This behavioral effect was supported by changes in an intracellular marker of DA neurotransmission [phosphorylated form of DARPP-32; pDARPP-32(Thr34)] in the striatum (López-Cruz et al., 2018). Similarly, theophylline reversed the effects induced by this D_2_ antagonist in mice tested in the T-maze barrier task (Pardo et al., 2012). Furthermore, several papers have reported that the adenosine A_2A_ receptor antagonist MSX-3 can reverse the effort-related effects of TBZ across multiple tasks (Nunes et al., 2013; Randall et al., 2014; Yohn et al., 2015). All these findings suggest that the reversal effects induced by methylxanthines on anergia induced by DA D_1_ and D_2_ receptor antagonism could be mediated mainly by A_2A_ receptors.

Mental fatigue associated with high attentional demands can also be overcome by the use of psychostimulants such as amphetamine or caffeine (Silber et al., 2006; Peeling and Dawson, 2007). For instance, caffeine restores memory performance in sleep-deprived or aged humans, a finding replicated in rodent animal models (Cunha and Agostinho, 2010). In cost/benefit decision-making tasks involving the evaluation of the costs related to high attention-demands, rats can choose between engaging in hard trials (difficult visuospatial discrimination) leading to more reward versus easy trials leading to less reward (Cocker et al., 2012). Under basal conditions, animals chose high effort/high reward trials more than low-effort/low reward trials. However, there are substantial baseline differences. Amphetamine increases the selection of high effort/high reward trials in animals that usually do not choose this option, but it decreases the selection of the high cognitive demand trials in animals that usually choose them. A high dose of caffeine decreased choice of high effort/high reward trials in animals that usually choose them as did amphetamine, but it did not increase the selection in the ones that usually did not choose them (Cocker et al., 2012).

## A_2A_ Receptor Antagonists have Therapeutic Actions on Cytokine-Induced Fatigue

Cytokines are signaling molecules for the immune system mediating physiological responses to infection (Dantzer, 2001). These molecules also mediate a set of behavioral signs that include depressed activity and loss of interest or motivation (Kent et al., 1992). Compared to the general population, depressed patients have elevated levels of proinflammatory cytokines such as tumor necrosis factor alpha (TN-alpha) interleukin-1β (IL-1β), and IL-6 (Dowlati et al., 2010; Hiles et al., 2012). Fatigue, loss of energy and psychomotor slowing are reported to occurred in patients receiving treatment with IFN-α or with high levels of IL-6 (Miller et al., 2009; Goldsmith et al., 2016b). Moreover, many inflammatory stimuli have been found to target reductions in ventral striatal neural function, and decreased synthesis of striatal DA, which is possibly related to symptoms of reduced motivation and motor retardation (Felger and Treadway, 2017). Studies with IL-6 indicate that this cytokine is responsive to stress, and is implicated in the production of depression-like effects in mice, including actions on traditional tests such as the FST, TST, and social interaction tests (Sukoff Rizzo et al., 2012). In anergia related studies, IL-6 and IL-1β reduced the tendency to work for food when an alternative food source (concurrently available chow) could be obtained through minimal effort (Nunes et al., 2014; Yohn et al., 2016a).

Brain cytokine signaling involves adenosine signaling at adenosine A_2A_ receptors (Hanff et al., 2010). These receptors regulate IL-1β and LPS linked to pathological behavioral and physiological responses such as anxiety (Chiu et al., 2014) or neuroinflammation (Brothers et al., 2010; Simões et al., 2012). Adenosine A_2A_ receptor signaling provides inhibitory feedback on proinflammatory cytokine signaling in peripheral immune cells (Sitkovsky and Ohta, 2005). Thus, the effects of IL-6 and IL-1β were attenuated through co-administration of the adenosine A_2A_ receptor antagonist MSX-3, as well as the major stimulant methylphenidate, which blocks catecholamine uptake (Nunes et al., 2014; Yohn et al., 2016c). Though previous work has shown that MSX-3 had no effect of FR5/chow-feeding choice performance when administered on its own (Farrar et al., 2007), MSX-3 produced a very robust reversal of the behavioral effects of IL-6 and IL-1β, restoring the baseline behavioral pattern of responding (i.e., increasing lever pressing and decreasing chow consumption) to a normal level (Nunes et al., 2014; Yohn et al., 2016c). These results highlight the therapeutic potential of adenosine A_2A_ receptor antagonism for pathologies related to neuroinflammation (Simões et al., 2012; Cunha, 2016).

## Anergia and Fatigue Influence Decision-Making in Humans with Depression

Translational studies in humans have implemented tasks that evaluate the decision-making process in normal as well as psychiatric patients. The effort expenditure for rewards task (EEfRT; Treadway et al., 2009), is based on the operant lever pressing choice tasks described above (Salamone et al., 1991). In the human version of this task, subjects choose on each trial between a high cost/high reward option (HC/HR) and low cost/low reward option (LC/LR) to obtain different monetary rewards. The HC/HR trials required 100 button presses with the non-dominant pinky finger within 21 s, and subjects were eligible to win higher amounts that varied per trial between $1.24–4.30. In contrast, the LC/LR option only required 30 button presses with the dominant index finger during 7 s, and subjects could win $1.00 for each successfully completed trial.

Patients with MDD were significantly less likely to make HC/HR choices relative to controls, and this result was not related with depression-related differences in psychomotor speed (Treadway et al., 2012). The effect of caffeine on this task in depressed patients has not been explored, but it was assessed in normal subjects. Thus, in the normal population, caffeine (200 mg), significantly increased the speed of responses compared to placebo (Wardle et al., 2012). However, caffeine did not have an effect on percentage of HC/HR choices (Wardle et al., 2012). In fact, it decreased effortful choices in high cardiovascular responders (subjects with high arterial pressure in response to caffeine) (Wardle et al., 2012). These results contrast with studies showing that, during exercise, caffeine decreases the perception of effort in humans (Doherty and Smith, 2005), improving performance particularly for endurance testing (Doherty and Smith, 2004). Thus, caffeine may only improve performance in highly demanding situations.

## Conclusion and Further Directions

Although many available treatments for MDD provide relief for individuals with depressed mood, no single therapeutic modality provides a full and permanent recovery across all the symptoms of MDD in the majority of patients (McClintock et al., 2011). Clinicians have come to emphasize the importance of taking into account effort-related motivational symptoms in depression (Tylee et al., 1999; Stahl, 2002; Demyttenaere et al., 2005; Salamone et al., 2016). Decreased psychomotor speed, referred to clinically as psychomotor retardation, fatigue and anergia are cardinal symptoms of MDD that have been associated with poor antidepressant treatment response (Goldsmith et al., 2016a). Even among patients in remission, anergia and psychomotor retardation are pervasive symptoms (Gorwood et al., 2014). Thus, novel pharmacological targets are being investigated in clinical and preclinical studies.

There are promising results shown in epidemiological studies as well as in animal models, about the impact of caffeine and selective adenosine receptor antagonists on these symptoms. Is worth noting that the epidemiological studies have revealed a relation between caffeine consumption and decreased risk for developing depression (Lucas et al., 2011), and some reports demonstrate the use of caffeine as a self-medication among depressed patients (Leibenluft et al., 1993). However, it seems clear that more controlled studies are needed to explore the effect of caffeine across a wide variety of depressive symptoms, and it seems necessary to test more selective drugs for A_2A_ receptors.

Systematic studies of the effects of methylxanthines on animal models of depression and anergia have shown efficacy at improving parameters related with initiation and maintenance of behavior in order to escape an aversive situation, but also in order to pursue valued reinforcers and achieve goals (Kulkarni and Mehta, 1985; Woodson et al., 1998; Hunter et al., 2003; Minor et al., 2008; Randall et al., 2011; Pechlivanova et al., 2012). As with the human data, these therapeutic actions depend upon the dose administered, since high doses of caffeine and theophylline not only do not improve depressive symptoms, but can in fact promote anxiety (Correa and Font, 2008; López-Cruz et al., 2014). Moreover, it is important to take into consideration that the use of high doses of caffeine and other methylxanthiness, specially, among the elder, could also have severe side effects such as tachycardia, gastric discomfort, or insomnia (Frozi et al., 2018). All these side effects could in fact worse the symptoms of MDD. Both in humans and in animal studies, the therapeutic actions of methylxanthines also seem to be dependent on the basal estate; for instance they seem to be effective when subjects are in a state or fatigue, tiredness or sleepiness (Johnson et al., 1990a; Smith et al., 1992; Childs and de Wit, 2008), or when the DArgic system is compromised. Such effects are less evident when humans and rodents are under “normal” conditions.

Several A_2A_ selective receptor antagonists have also shown to reverse motivational impairments induced by DA antagonism or depletion in animal models of anergia (Farrar et al., 2007; Mott et al., 2009; Salamone et al., 2009; Pardo et al., 2012; Correa et al., 2016). Furthermore, a recent report indicates that istradefylline can improve fatigue-related symptoms in Parkinson’s disease patients (Abe et al., 2016; Sako et al., 2017). Adenosine A_2A_ receptors might be involved in these processes through their interaction with DA D_2_ receptors in the Acb, region highly involved in the activational component of motivation (for a review see Salamone and Correa, 2012).

Consistent with these findings, it has been demonstrated that the rank order of clinical effectiveness in depressed patients with psychomotor retardation, paralleled the specificity of antidepressants as DA-mimetic agents (Rampello et al., 1991). Antidepressants such as bupropion have demonstrated to have therapeutical effects on motivational symptoms in humans (Pae et al., 2007) and in animal models or anergia (Nunes et al., 2013; Randall et al., 2014; Yohn et al., 2015). In animal studies, caffeine was shown to improve the effects of antidepressants such as bupropion, duloxetine, and desipramine (Robles-Molina et al., 2012; Kale and Addepalli, 2014; Kale and Addepalli, 2015; Szopa et al., 2016). These studies have led to the suggestion that caffeine could be used as an enhancer of antidepressant pharmacotherapy (for a review see Kale et al., 2010), a suggestion that is consistent with the clinical trials for antiparkinsonian effects showing that A_2A_ receptor antagonists can be a good adjuvant in the treatment of motor symptoms (Hung and Schwarzschild, 2014).

However, determination of the predominant symptomatology is key to therapeutic success. Recent neuroimage data from patients with depression indicate that they can be clustered based on four different connectivity profiles (‘biotypes’) that are associated with differences in clinical symptoms (Drysdale et al., 2017). Thus, reduced connectivity in anterior cingulate and orbitofrontal areas supporting motivation was most severe in biotypes 1 and 2, which were characterized partly by increased anergia and fatigue (Drysdale et al., 2017). This type of objective diagnostic can help to identify different type of patients that could benefit from different type of antidepressant therapies. For instance, in patients affected by anxious depression a selective inhibitor of 5-HT reuptake appears to be more effective than a selective inhibitor of DA reuptake (Rampello et al., 1995), and caffeine in those type of depressed patients may worsen the anxiety symptomatology. However, adenosine A_2A_ receptor antagonism may offer an alternative therapeutic strategy for treating effort-related motivational dysfunctions in humans, probably with lower abuse liability and fewer major stimulant motor effects compared to DA uptake inhibitors.

## Author Contributions

All authors listed have made a substantial, direct and intellectual contribution to the work, and approved it for publication.

## Conflict of Interest Statement

JS has received grants from Merck-Serrono, Pfizer, Roche, Shire, and Prexa. MC has received a grant from Servier. The other author declares that the research was conducted in the absence of any commercial or financial relationships that could be construed as a potential conflict of interest.
